# Enhancing and Tuning the Nonlinear Optical Response and Wavelength-Agile Strong Optical Limiting Action of *N*-octylamine Modified Fluorographenes

**DOI:** 10.3390/nano10112319

**Published:** 2020-11-23

**Authors:** Aristeidis Stathis, Michalis Stavrou, Ioannis Papadakis, Ievgen Obratzov, Stelios Couris

**Affiliations:** 1 Department of Physics, University of Patras, 26504 Patras, Greece; a.stathis@iceht.forth.gr (A.S.); m.stavrou@iceht.forth.gr (M.S.); j.papadakis@iceht.forth.gr (I.P.); 2 Institute of Chemical Engineering Sciences (ICE-HT), Foundation for Research and Technology-Hellas (FORTH), P.O. Box 1414, 26504 Patras, Greece; 3 Regional Centre of Advanced Technologies and Materials, Faculty of Science, Palacky University in Olomouc, 77900 Olomouc, Czech Republic; ievgen.obraztsov@upol.cz

**Keywords:** *N*-doped graphene, nonlinear optical response, optical limiting, graphene functionalization

## Abstract

Fluorographene has been recently shown to be a suitable platform for synthesizing numerous graphene derivatives with desired properties. In that respect, *N*-octylamine-modified fluorographenes with variable degrees of functionalization are studied and their nonlinear optical properties are assessed using 4 ns pulses. A very strong enhancement of the nonlinear optical response and a very efficient optical limiting action are observed, being strongly dependent on the degree of functionalization of fluorographene. The observed enhanced response is attributed to the increasing number of defects because of the incorporation of N-heteroatoms in the graphitic network upon functionalization with *N*-octylamine. The present work paves the way for the controlled covalent functionalization of graphene enabling a scalable access to a wide portfolio of graphene derivatives with custom-tailored properties.

## 1. Introduction

In recent years, fluorographene [1,2] (FG) has become a template for the synthesis of new members of the graphene family [3,4,5], with controllable chemical and optical properties, suitable for a plethora of applications such as biosensing [6], gas sensing on surfaces [7] and solar cell technologies [8]. Unlike its parent, graphene, FG possesses great chemical activity because of the electronegative fluorine that attaches to carbon atoms along with the formation of various structural defects, placing FG among the suitable carbon precursors for the tunable and scalable synthesis of new graphene derivatives with desired properties [9,10]. Recent examples include thiol-, amine- and hydrogenated derivatives obtained by the partial nucleophilic substitution of FG by organic thiophenols (methoxy-thiophenol, dimethyl-amin-thiophenol), lithium diethylamide and H-, respectively [11,12,13]. The newly synthesized derivatives contain the selected ligand via substitution, resulting in the formation of sp^2^ nanodomains arising from the fluorine atoms’ elimination, while they also contain some residual carbon-fluorine groups. Herein, we investigate the effect of the amine functionalization of FG via *N*-octylamine nucleophilic substitution on its nonlinear optical (NLO) response and optical limiting (OL) performance under nanosecond laser excitation. Doping FG with heteroatoms, such as nitrogen, can significantly alter its electronic structure due to tailored defect-induced states, leading to novel 2D materials whose optical and electronic properties can be controlled by modifying the amount of nitrogen (%N) incorporated into the graphenic lattice. For instance, nitrogen doping can tune the electrical properties of some chemically functionalized graphenes, e.g., from a p-type semiconductor [14] to an n-type one [15]. In the present work, four FG derivatives, namely FG-OAx, with different F and N contents (ranging from 55% down to 2.3%, and from 2.9% up to 8.1%, respectively), were synthesized in order to demonstrate and quantify the effects of such functionalization on the NLO properties and OL action of the aforementioned derivatives. The atomic F and N content (F/N) was controlled by tweaking the reaction time of the *N*-octylamine (OA) ligand with FG. The investigated FG derivatives exhibited enhanced NLO responses compared to pristine FG and other covalently functionalized graphene derivatives, demonstrating the importance and effectiveness of graphene functionalization for superior NLO response and its tunability. As far as the OL action is concerned, the present derivatives were found to exhibit sizeable OL action in the range of 450 to 1950 nm, operating more efficiently for visible wavelengths. The OL action of these FG-OAx derivatives arises mainly from their strong nonlinear absorption (NLA), which is significantly affected by the tailored defects of the derivatives. The present study demonstrates clearly that the heteroatom doping of FG is an efficient tool for the control of the NLO properties through controlled inducing defects. The further exploitation of these defect-engineered FGs can lead to materials suitable for various photonic and optoelectronic applications.

## 2. Materials and Methods

### 2.1. Synthesis and Characterization

Graphite fluoride, GrF (>61 wt. % F, C1F1.1), was purchased from Sigma-Aldrich (St. Louis, MO, USA) and was used for the synthesis of pristine FG. The latter was obtained by the exfoliation of GrF in *N*,*N*-Dimethylformamide, or namely, DMF [16]. The four FG-OAx derivatives were prepared by partial nucleophilic substitution with the corresponding *N*-octylamine ligand, as previously described [17,18]. The alkylamine ligand acted as a nucleophile, attacking the electrophilic radical centers of FG, according to the recently explained pathway of the reactions of nucleophiles with FG [19] The reaction led to the covalent attachment of the octylamine molecules to the graphenic plane. The investigated samples were characterized by an arsenal of techniques, such as X-ray Photoelectron Spectroscopy (XPS),**** Fourier-transform infrared spectroscopy (FT-IR), Transmission Electron Microscopy (TEM) and Raman spectroscopy, to determine and confirm the successful functionalization of FG. The detailed synthesis along with the characterization results can be found in detail in references [17,18]. The XPS results have been previously reported and are presented here for the sake of better understanding in the context of explaining the photophysical properties. The four (*N*-octylamine)-functionalized FG derivatives were named as FG-OA 20′, FG-OA 30′, FG-OA 6 h and FG-OA 24 h, where the numbers indicate the reaction time. Raman spectra were acquired using a Dynamic Extended Resolution (DXR) Raman spectroscope (Thermo Scientific, Waltham, MA, USA) equipped with a laser operating at a wavelength of 633 nm. The dried powder sample was deposited on the substrate, and the laser power on the sample was set to 2 mW and the exposure time was 2 s. Each measured Raman spectrum represents an average of 1024 experimental microscans.

### 2.2. Experimental Setups for the Measurements of the NLO Properties and the OL Performance

The non-linear optical (NLO) response of the FG-octylamine (FG-OAx) derivatives was investigated by means of the Ζ-scan technique [20], employing visible (532 nm) and infrared (1064 nm) 4 ns laser pulses. The Z-scan technique was selected because of its experimental simplicity and because it allows the simultaneous determination of both the magnitude and the sign of the nonlinear absorption, and the refraction of a sample from a single measurement. The nonlinear absorption and refraction of a sample are expressed by the nonlinear absorption coefficient β and the nonlinear refractive parameter γ′, which are related to the imaginary, *Imχ*^(3)^, and real, *Reχ*^(3)^, parts of the third-order nonlinear susceptibility, *χ*^(3)^. A detailed description of the experimental setup as well as the procedures followed for the analysis of the Z-scans data can be found in detail elsewhere [21]. For the Z-scan experiments, a 4 ns Q-switched Nd:YAG laser (EKSPLA NT 342/3/UVE/AW) was employed, operating at a repetition rate between 1 and 10 Hz, at its fundamental (i.e., at 1064 nm) or at its second harmonic frequency (i.e., at 532 nm). For the optical limiting experiments, an Optical Parametric Oscillator (OPO) was used, pumped by the same Nd:YAG laser. In all cases, the laser beam was focused into the samples using a 20 cm focal length quartz plano-convex lens. Several dispersions with appropriate concentrations in order to exhibit the same linear transmittance, i.e., of ~70%, at each excitation wavelength used (i.e., at 450, 532, 650, 750, 950, 1064, 1250, 1550 and 1750 nm) were prepared. The corresponding spot radii at the focus were determined using a charge-coupled device (CCD) camera, to achieve the incident laser intensity and/or the laser fluence needed for the experiments. The prepared dispersions of each FG-octylamine derivative were placed in 1 mm thick quartz cells for the optical measurements. For the Z-scan measurements performed at 532 and 1064 nm, the corresponding Rayleigh length (i.e., confocal parameter) was determined. They were about 2 and 2.6 mm, respectively, thus fulfilling the Z-scan condition concerning the thin sample approximation. For comparison purposes, some C_60_ toluene solutions were also prepared and used as reference during the evaluation of the OL performance, since C_60_ is generally considered as a benchmark material for OL studies.

## 3. Results and Discussion

### 3.1. Measurements of the NLO Properties

**Figure 1:**
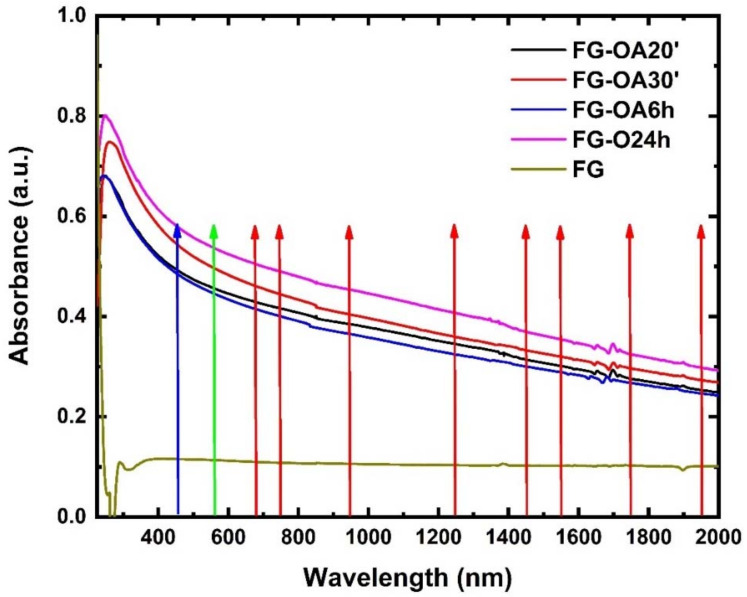
UV-Vis-NIR absorption spectra of pristine FG and the FG-OAx derivatives (all dispersed in DCM).

Some representative UV-Vis-NIR absorption spectra of FG and the four octylamine-functionalized FGs (FG-OAx), all dispersed in dichloromethane (DCM), are presented in Figure 1. As shown, FG exhibits the typical graphene featureless structure, whereas the spectra of the FG-OAxs exhibit an increasing absorption towards shorter wavelengths and a clearly distinct absorption feature between 250 and 265 nm, ascribed to the sp^2^-conjugated carbons emerging from the reductive defluorination of FG [22,23]. The colored arrows indicate schematically the excitation wavelengths where the optical limiting (OL) action of the dispersions was evaluated.

**Figure 2:**
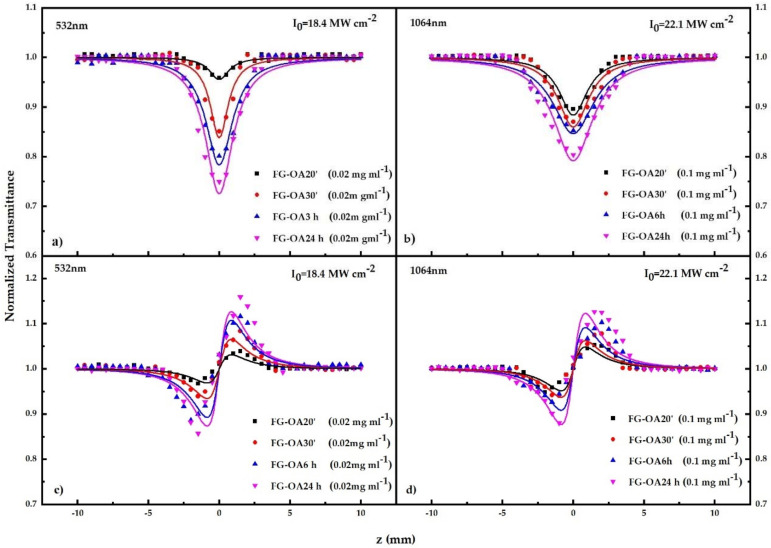
(**a**,**b**) “Open-aperture” (OA) and (**c**,**d**) “divided” (CA) Z-scans obtained under 4 ns 532 and 1064 nm laser excitations.

For the determination of the nonlinear optical parameters (i.e., β and γ′) of the investigated FG-OAxs, both “open-” (OA) and “closed-aperture” (CA) Z-scan measurements were performed using FG-OAx dispersions of various concentrations, ranging from 0.01 to 0.4 mg ml^−1^. The measurements were conducted under several incident laser intensities (ranging from 0.86 to 111 MW cm^−2^). The corresponding “divided” Z-scans were prepared. The OA and the “divided” Z-scans were used to deduce the nonlinear absorption parameter β and the nonlinear refractive index parameter γ′, respectively, of the sample. In Figure 2, some representative OA and “divided” Z-scan recordings obtained under 4 ns 532 nm (a,c) and 1064 nm (b,d) laser excitation are presented. Since the solvent (i.e., DCM) exhibits a negligible NLO response under the present experimental conditions, the shown “open-“and “divided” Z-scan recordings of Figure 2 reveal directly the NLO response of the FG-OAxs. All OA Z-scan curves exhibited a transmission minimum, indicative of reverse saturable absorption (RSA) behavior, corresponding to a positive sign nonlinear absorption coefficient (*β* > 0 or *Imχ*^(3)^ > 0). In fact, all the FG derivatives were found to exhibit RSA behavior, under all the experimental conditions used (i.e., incident laser intensity and dispersion concentrations), in both the visible and infrared excitation regimes. In principle, such an RSA response can be due to two-photon absorption (2PA) or excited state absorption (ESA) [24]. Further discussion about the origin of the RSA behavior is presented below the optical limiting action results.

The magnitude and the sign of the nonlinear refractive index parameter *γ′* were determined from the corresponding “divided” Z-scans. All FG-OAxs were found exhibiting a valley–peak transmission configuration, thus indicating the self-focusing behavior corresponding to *γ′* > 0, or equivalently to *Reχ*^(3)^ > 0, under all the experimental conditions employed (Figure 2). In order to avoid the formation of cumulative thermal effects, all experiments were performed with a 1 Hz repetition rate of the laser.

Following the standard Z-scan data analysis procedures [25], the NLO parameters *β* and *γ*′ and the third-order nonlinear susceptibility *χ*^(3)^ were determined, and they are summarized in Table 1. To make comparisons easier, all the shown values refer to a concentration of 1 mg ml^−1^.

**Figure 3:**
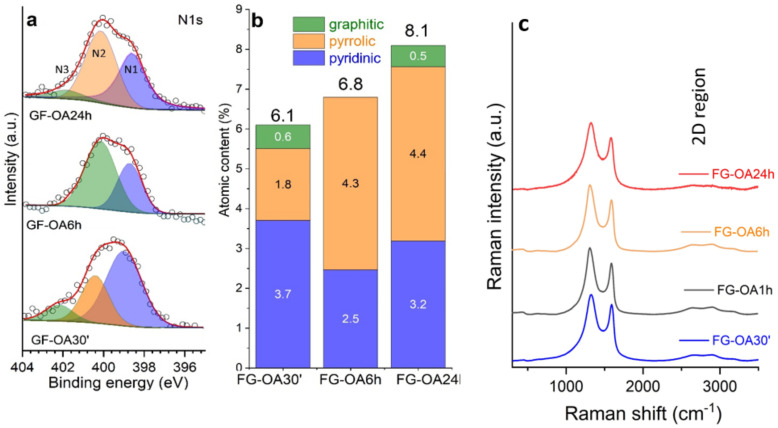
(**a**) High-resolution X-ray photoelectron spectroscopy of the N1s regions showing the deconvoluted nitrogen components in pyridinic (N1), pyrrolic (N2) and graphitic (N3) configurations. (**b**) Total atomic nitrogen content in the samples and atomic contents of the different nitrogen components. (**c**) Raman spectra.

Regarding the effect of the degree of FG functionalization on the nonlinear absorption of the FG-OAxs, a dramatic enhancement is observed as the reaction of FG with *N*-octylamine proceeds, at both excitation wavelengths, as can be seen from the evolution of the β (or Imχ^(3)^) values in Table 1. More specifically, the nonlinear absorption coefficient β was found to attain a maximum after 24 h of reaction, the FG-OA24 exhibiting an almost 100-fold increase compared to FG, for visible excitation. Interestingly, as far as concerns infrared excitation at 1064 nm, where FG exhibits insignificant NLO absorption, this enhancement was much more pronounced, attaining a 6300-fold increase. To understand the observed enhancement of the nonlinear absorption, the different bonding configurations that nitrogen creates in the graphenic lattice have to be considered [26]. The most usual bonding configurations reported are the following: the graphitic-N bonding, where an N atom bonds to three C atoms directly, eliminating one C atom; the pyridinic-N bonding, where a six-membered ring is formed as an N-atom is accompanied by a vacancy and bonds to two C atoms, and the pyrrolic-N bonding, where a nitrogen atom bonds to two carbon atoms formatting a five-membered ring. Among these bonding configurations, graphitic-N and pyridinic-N bondings are of *sp*^2^ hybridization, while the pyrrolic-N one is of *sp*^3^ hybridization. As reported by Zaoralová et al., DFT calculations showed that N incorporation into the carbon lattice can mostly occur at the several types of vacancies present in pristine FG, with nitrogen atoms assigned mainly in the case of pyridinic and pyrrolic configurations [12]. The presence of these configurations was further confirmed by high-resolution X-ray photoelectron spectroscopy. The corresponding results are presented in Figure 3a, where the deconvoluted N1s envelope is shown (pyrrolic 400 eV, pyridinic 398.5 eV). Figure 3b shows the total atomic nitrogen content in the samples and atomic contents of the different nitrogen components.

In Figure 3c the Raman spectra of the FG-OAxs are presented. As shown, all FG-OAxs exhibited broad D and G bands and a flat 2D region (around 2800–2900 cm^−1^). These findings suggest the presence of a large number of defects as well as functional groups, which disturb the hexagonal network of graphene. As a result, the pure graphenic sp^2^ domains are smaller in size, as supported by the observed suppression of the 2D band. In addition, as the time of reaction increases, a broadening of the D band of the FG-OAxs is observed, as can be seen in the Table 2 below. This evidence can be attributed to the increasing number of defects [27].

Based on the above experimental evidence, the observed enhancement of the NLA can be attributed to the large number of defect-induced states, created by the interplay between *sp*^2^/*sp*^3^ sites (pyrollic and pyridinic configurations). In addition to these arguments, the differences between the local band gaps of the *sp*^3^ and *sp*^2^ domains in the graphitic structures create band edge fluctuations, with the *sp*^3^ sites acting as tunnel barriers between the π states of the *sp*^2^ domains [28]. This situation results in the creation of strongly localized isolated *sp*^2^ sites, which can also act as defects in the electronic band. As such, summarizing, it seems that as the degree of functionalization increases with the reaction time elapsed (i.e., higher N atom content corresponding to lower F atom content), the observed NLA increases, due to the increasing number of defects present in the FG-OAxs.

Concerning the NLO refractive response of the FG-OAxs, an alteration of the sign of the nonlinear refractive index parameter *γ’* was observed between FG and the FG-OAxs, as can be seen in Table 1. This sign change can be understood by evoking the large difference in F atom content between the FG (i.e., 55%) and the FG-OAxs (ranging from 33% down to 2.3%), as has been discussed in detail elsewhere [29].

Another parameter which contributes to the NLO response modification of FG upon *N*-octylamine functionalization is related to the changing nature of the C–F bonds in these derivatives, due to the presence of the highly electronegative fluorine atoms. So, the nature of the C–F bond can vary within the FG lattice depending on the F content. Specifically, the C–F bond can be introduced in the form of covalent, ionic or semi-ionic ones, strongly depending on the F atom content. In fact, at low F atom contents, ionic C–F bonding is favored, whereas increasing the F atom content, semi-ionic and covalent C–F bondings prevail, as has been reported elsewhere [30]. Hence it is expected that in the present FG derivatives, both C–F bonding configurations should be present, varying with the F atom content, i.e., the degree of functionalization.

Summarizing the above, it can be concluded that increasing the time of reaction between the *N*-octylamine ligand and pristine FG, the NLO response of the FG-OAxs increases greatly, both under visible and infrared laser excitation, in the latter case observing the largest enhancement of the NLO response by a factor of about 6300 times compared to FG. Furthermore, it is important to remember that the chemical functionalization of FG has an ON/OFF switching effect on the NLO response to excitation with infrared laser pulses. It is interesting to recall that in previous reports [11] concerning the study of some thiophenol- and diethylamino-modified FGs, under similar experimental conditions, the resulting NLO response of the FG derivatives was found to be much weaker than that found in the present study for the FG-OAxs ones. This result reveals the importance of the type of functionalization ligand of FG for the enhancement and the tailoring of the NLO response of FG derivatives.

### 3.2. Optical Limiting Measurements

**Figure 4:**
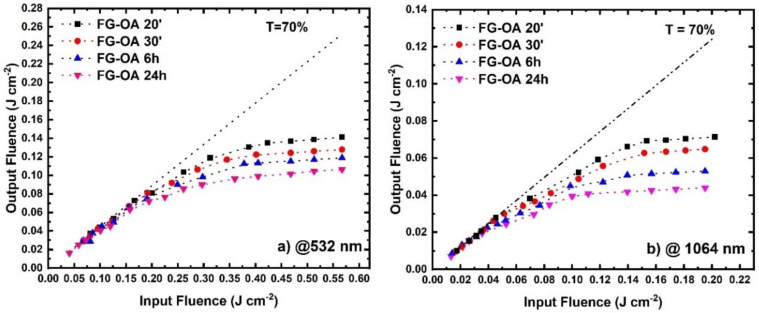
Optical limiting action of FG-OAxs under (**a**) 532 and (**b**)1064 nm laser irradiation.

**Figure 5:**
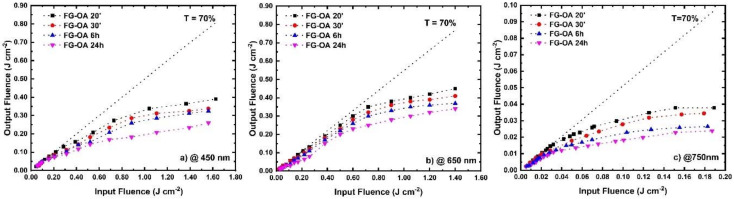
Optical limiting action of FG-OAxs under (**a**) 450, (**b**) 650 and (**c**) 750 nm laser irradiation.

**Figure 6:**
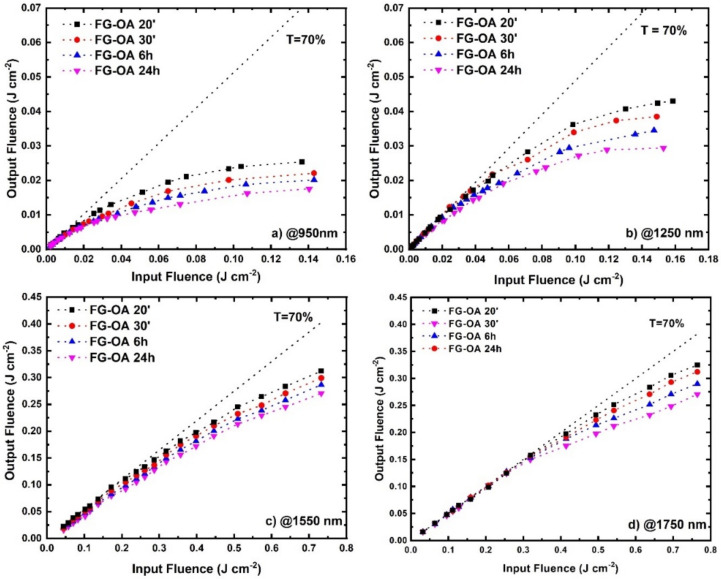
Optical limiting action of FG-OAxs under (**a**) 950, (**b**) 1250, (**c**) 1550 and (**d**) 1750 nm laser irradiation.

Next, the optical limiting (OL) action of the FG-OAxs was assessed under different wavelength laser excitations, extending from visible (i.e., 450, 532, 650, 750 nm) to NIR (i.e., 950, 1064, 1250, 1440, 1550 and 1750 nm) wavelengths. The colored arrows shown in Figure 1 indicate the wavelengths at which the OL action was studied. More specifically, the OL action of the FG-OAxs was evaluated by measuring the incident laser beam energy on the sample, the outgoing laser beam energy, and calculating the corresponding laser fluences, F_in_ and F_out_, respectively, after taking into account the laser beam spot size. The laser beam energies were measured using two calibrated joulemeters, while the size of the irradiating area was measured by a CCD camera. From the curves shown in Figure 4a,b, Figure 5a–c and Figure 6a–d, presenting the variation of the F_out_ versus F_in_, the optical limiting onset, OL_on_, was deduced. The OL_on_ is defined as the value of the input laser fluence, F_in_, at which the sample’s linear transmittance starts to deviate from the Beer–Lambert law. The dashed lines indicate the linear transmittance of the sample (e.g., at the far left/right wings of the OA Z-scans for 532/1064 nm excitation, and the F_out_/F_in_ ratio at low F_in_ fluence). The obtained OL action results, for each excitation wavelength and for all FG-OAxs, are presented in Figure 4 and Figure 5. As can be seen in these figures, all *N*-octylamine functionalized FGs exhibited a very important OL action, which was found to be increasing with the degree of functionalization (i.e., the reaction time between FG and *N*-octylamine). In Figure 4a,b, the OL actions of the FG-OAxs under the 532 and 1064 nm laser excitations are presented separately from the OL action studied at the other excitation wavelengths used, as the results concerning the 532 and 1064 nm excitation wavelengths are the most commonly reported in the related literature. The corresponding OL_on_ values for each FG-OAx, determined from the curves of Figure 4 and Figure 6, are presented in Table 3. It is important to remember that lower OL_on_ values indicate more efficient OL action. From the monotonically decreasing OL_on_ values of Table 3, it becomes evident that as the reaction time of FG with *N*-octylamine increases, the optical limiting efficiency is greatly improved. This is attributed to the increasing number of defects, as discussed previously, leading to more important NLA, which is the principal mechanism responsible for the observed OL action exhibited by the FG-OAxs, as will be discussed in more detail in the following.

As can be seen form this table, the OL_on_ values of the FG-OAxs (all dispersions prepared exhibiting 70% linear transmittance at each excitation wavelength) were all found to be quite low, both under visible and infrared excitation, and in the latter case being substantially smaller. This finding suggests clearly that the FG-OAxs are much more efficient optical limiters when compared, for example, to other materials generally considered as benchmark materials for optical limiting, e.g., some C_60_-toluene solutions (with OL_on_ = 0.2 J cm^−2^) [31] and carbon black suspensions (with OL_on_ = 2.2 J cm^−2^) [32].

Then, the optical limiting action of the FG-OAx derivatives was investigated under irradiation by laser radiations of wavelengths ranging from the visible to the NIR spectral region, i.e., 450, 650, 750, 950, 1250, 1550 and 1750 nm. In Figure 5 and Figure 6, the obtained OL action of the FG-OAxs under the different irradiation wavelengths is presented. In all cases, efficient OL action was observed, becoming more efficient from 450 nm to about 1250 nm, while becoming less efficient towards the NIR wavelengths. The corresponding OL_on_ values determined from the OL curves of Figure 5 and Figure 6 are listed in Table 4.

**Figure 7:**
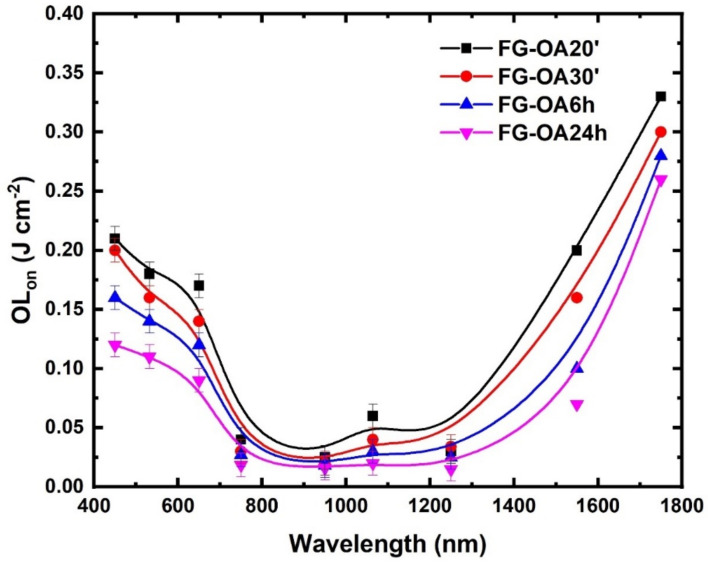
Variation of the optical limiting onset (OL_on_) of the FG-OAx derivatives with the irradiation wavelength.

The variation of the OL_on_ values from visible to NIR wavelengths is schematically depicted in Figure 7. As shown, the OL_on_ values of all FG-OAxs were found to be decreasing towards 800 nm, attaining a plateau in the spectral region from about 750 to 1250 nm, then again increasing towards 1750 nm. Specifically, the OL_on_ values of the studied FG-OAxs were found decreasing by almost an order of magnitude, attaining very low values of about 0.01 J cm^−2^ at 950 and 1250 nm.

Compared to other 2D nanomaterials, the studied FG-OAxs are found to exhibit a much stronger OL behavior. So, in a recent work [33] studying the OL of graphene and some transition metal dichalcogenides-TMDCs (e.g., MoS_2_, MoSe_2_, WS_2_, and WSe_2_) nanosheet dispersions under 6 ns 532 and 1064 nm laser excitations, it was reported that graphene exhibited the lowest onset of OL, i.e., ~0.44 and ~0.64 J cm^−2^, respectively, whereas the TMDC dispersions exhibited OL_on_ values larger than 1 and 1.37 J cm^−2^, respectively. In another work [34], concerning the OL action of some graphene nanosheets (GNS), graphene nanoribbons (GNR), graphene oxide nanosheets (GONS) and graphene oxide nanoribbons (GONR), it was found that all these derivatives exhibited OL_on_ values ranging between 0.5 and 3 J cm^−2^ for visible excitation, whereas for infrared excitation the OL_on_ was found to be significantly larger, i.e., larger than 3.5 J cm^−2^. So, in all cases, the FG-OAxs studied here exhibited much more effective optical limiting.

Regarding the OL action of some other fluorographene derivatives studied by our group, such as some thiophenol-modified fluorographenes [11], the present *N*-octylamine-modified fluorographenes were found to exhibit comparable OL action. More specifically, the thiophenol fluorographenes exhibited OL_on_ values of ~0.1 J cm^−2^ and ~0.05 J cm^−2^ under 532 and 1064 nm excitations, respectively, which are similar to the OL_on_ values of FG-OA 24 h and FG-OA 6 h derivatives. In another work [13] studying the OL action of a diethylamino-modified fluorographene (CDEA), it was found that its OL_on_ was increasing towards the NIR wavelengths. In particular, the OL_on_ of CDEA was found to be ~0.045 J cm^−2^ at 532 nm, increasing continuously and reaching the value of ~0.16 J cm^−2^ at 1750 nm. So, in this case, the present FG-OAxs exhibit slightly weaker OL values at both 532 and 1750 nm. However, the FG-OAxs were found to exhibit significantly stronger OL values in the region 750–1250 nm, with their OL_on_ being over an order of magnitude lower than that of CDEA.

In general, there are different processes that can give rise to an optical limiting action. Some of the processes occurring under ns laser irradiation include the nonlinear absorption (NLA), the nonlinear scattering (NLS) and the induced thermal scattering (ITS). As far as concerns the last two processes, separate measurements were performed to evaluate their contributions to the observed OL action. So, at first, the presence of NLS was examined. In that view, experiments were conducted whereby the sample was irradiated by the laser beam while a sensitive photodiode was placed behind it, positioned at different directions with respect to the laser beam propagation direction. In front of the photodiode, a slit was placed to minimize the light due to reflections of the cell walls, ambient light, etc. However, for the range of laser energies used, negligible NLS was detected for all FG-OAx samples.

Next, the presence of scattered light originating from ITS-related phenomena has been explored. In this case, the formation of scattering centers (e.g., bubbles) in the sample due to heating of the surrounding medium can occur. As it has been reported elsewhere (e.g., [35,36]), ns laser pulses can result in the efficient heating of the graphene flakes. This excess heating can be efficiently dissipated to the surrounding solvent, giving rise to the formation of solvent bubbles, which expand quickly at the vapor–solution interface, acting as efficient scattering centers. In addition to this, powerful enough laser beams can induce micro-plasmas, which can also operate as scattering centers. To investigate such ITS phenomena in the present measurements, the unfocused beam of a Helium–Neon (He-Ne) laser was used, crossing the focal volume of the focused Nd:YAG laser beam. The projection of the He-Ne beam onto a white screen placed a few meters away from the sample allows the visual observation of bubble formation. Although no such phenomena were observed for the laser energies employed in the present study, for precaution reasons, all experiments were carried out with operating the lasers at a 1 Hz repetition rate.

Finally, the mechanism responsible for the nonlinear absorption of the studied FG-OAxs was investigated. As mentioned in the previous section, for the Z-scan measurements carried out under 532 and 1064 nm excitations, they were all found exhibiting valley-shaped “open-aperture” Z-scans, corresponding to RSA behavior, implying the presence of nonlinear absorption. However, depending on the excitation wavelength, the pulse duration and the material properties, the observed nonlinear absorption can arise from two-photon absorption (2PA) and/or excited state absorption (ESA). To evaluate the nonlinear absorption coefficient *β*, the OA Z-scans were fitted by the following equation, described in detail elsewhere [37]:

(1) $$T\left(x\right)=\frac{1}{\sqrt{\pi }\left(\frac{\beta I_{0}L_{eff}}{1+{\left[x\right]}^{2}}\right)}\int_{-\infty }^{+\infty }\ln\left[1+\frac{\beta I_{0}L_{eff}}{1+{\left[x\right]}^{2}}e^{-t}\right]dt$$

This equation implies nonlinear absorption due to two-photon absorption (2PA). As can be seen from the solid lines of the OA Z-scan curves, corresponding to the best fit by this equation, a very successful fitting was obtained, suggesting that the 2PA process is the main mechanism giving rise to the RSA behavior.

**Figure 8:**
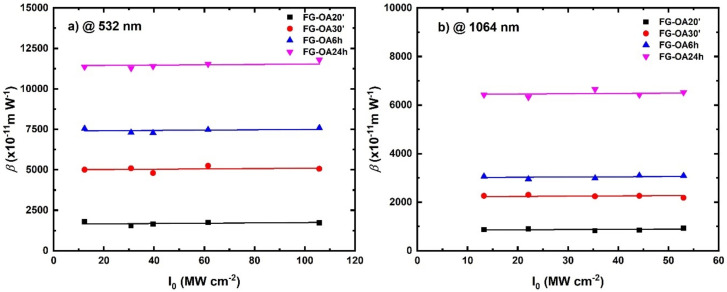
Dependence of the nonlinear absorption coefficient β upon the on-axis peak intensity for (**a**) 532 nm and (**b**) 1064 nm laser excitation.

Furthermore, the determined nonlinear absorption coefficient, *β*, was plotted as a function of the on-axis peak intensity for all FG-OAxs. If the ESA process is operating, it should cause the depletion of the ground state, leading to variations of the β with the on-axis peak intensity [38]. However, as illustrated in Figure 8a,b, the *β* was found to be independent of the on-axis peak intensity, under both the 532 and the 1064 nm excitation, thus suggesting that any contribution of the ESA to the nonlinear absorption should be negligible. Therefore, it can be safely concluded that 2PA seems to be the principal mechanism of the observed OL action.

## 4. Conclusions

In conclusion, pristine fluorographene (FG) and four FG derivatives (FG-OAx), prepared after the partial nucleophilic substitution of FG with *N*-octylamine and having different degrees of functionalization, were studied. The NLO responses of the FG and the FG-OAx derivatives were investigated by means of the Z-scan technique, along with their OL action, under various excitation wavelengths ranging from visible (450 nm) to NIR (1750 nm). Their NLO response was turned ON shortly after the initiation of the partial nucleophilic substitution of the FG, leading to strong enhancement with the time of reaction. The FG-OAxs were found to be exhibiting strong nonlinear absorptions, attributed to defect-induced states due to the C–N bonding configuration formed by the incorporation of nitrogen into the graphenic lattice. Their strong nonlinear absorption was found to result in extremely efficient OL action, rendering the FG-OAxs very efficient optical limiters suitable to and attractive for a plethora of OL applications, from the visible to NIR spectral regions. The above experimental findings demonstrate clearly that FG is a useful platform for achieving chemical functionalization, leading to a wide portfolio of fluorographene derivatives, while by controlling the degree of functionalization the nonlinear optical properties of the derivatives can be largely tuned, providing materials with tailored properties throughout almost the entire optical spectrum, which are highly desirable for several photonic and optoelectronic applications.

## Figures and Tables

**Table 1:** NLO parameters of the different octylamine-functionalized FGs.

Wavelength (nm)	Sample	Β (×10^−11^ m W^−1^)	γ′ (×10^−18^ m^2^ W^−1^)	Imχ^(3)^ (×10^−13^ esu)	Reχ^(3)^ (×10^−13^ esu)	|χ|^(3)^ (×10^−13^ esu)
532 nm	FG	119 ± 19	−135 ± 31	62 ± 12	−173 ± 38	184 ± 40
	FG-OA 20′	1720 ± 175	1325 ± 235	900 ± 95	1695 ± 300	1920 ± 315
	FG-OA 30′	5010 ± 1050	1680 ± 230	2640 ± 550	2120 ± 460	3380 ± 720
	FG-OA 6 h	7550 ± 1300	4275 ± 425	3950 ± 650	5475 ± 550	6725 ± 770
	FG-OA 24 h	11,363 ± 663	4252 ± 600	5950 ± 350	5800 ± 775	8325 ± 856
1064 nm	FG	-	-	-	-	-
	FG-OA 20′	872 ± 55	530 ± 100	943 ± 59	676 ± 128	1160 ± 142
	FG-OA 30′	2263 ± 345	1193 ± 198	2448 ± 372	1523 ± 350	2885 ± 450
	FG-OA 6 h	3063 ± 200	2648 ± 200	3313 ± 250	3350 ± 250	4713 ± 475
	FG-OA 24 h	6425 ± 388	3475 ± 330	6950 ± 425	4575 ± 725	8313 ± 750

**Table 2:** Broadening of the D band of FG-OAxs.

Sample	I_D_ cm^−1^	FWHM I_D_ cm^−1^
30 min	1325	103
1 h	1316	106
6 h	1315	109
24 h	1330	118

**Table 3:** Optical limiting onsets (OL_on_) of FG-OAxs under 532nm and 1064 nm laser irradiation.

Wavelength (nm)	Sample	OL_on_ (J cm^−2^)
532	FG-OA 20′	0.18
	FG-OA 30′	0.16
	FG-OA 6 h	0.13
	FG-OA 24 h	0.10
1064	FG-OA 20′	0.06
	FG-OA 30′	0.04
	FG-OA 6 h	0.03
	FG-OA 24 h	0.02

**Table 4:** Optical Limiting onsets of FG-OAxs under 450, 650, 750, 950, 1250, 1550 and 1750 nm laser irradiation.

Wavelength (nm)	Sample	OL_on_ (J cm^−2^)
450	FG-OA 20′	0.21
	FG-OA 30′	0.20
	FG-OA 6 h	0.15
	FG-OA 24 h	0.12
650	FG-OA 20′	0.17
	FG-OA 30′	0.14
	FG-OA 6 h	0.12
	FG-OA 24 h	0.09
750	FG-OA 20′	0.04
	FG-OA 30′	0.03
	FG-OA 6 h	0.03
	FG-OA 24 h	0.02
950	FG-OA 20′	0.02
	FG-OA 30′	0.02
	FG-OA 6 h	0.02
	FG-OA 24 h	0.01
1250	FG-OA 20′	0.03
	FG-OA 30′	0.03
	FG-OA 6 h	0.02
	FG-OA 24 h	0.01
1550	FG-OA 20′	0.20
	FG-OA 30′	0.16
	FG-OA 6 h	0.10
	FG-OA 24 h	0.07
1750	FG-OA 20′	0.33
	FG-OA 30′	0.30
	FG-OA 6 h	0.28
	FG-OA 24 h	0.26
